# Growth inhibition of thyroid follicular cell-derived cancers by the opioid growth factor (OGF) - opioid growth factor receptor (OGFr) axis

**DOI:** 10.1186/1471-2407-9-369

**Published:** 2009-10-18

**Authors:** Patricia J McLaughlin, Ian S Zagon, Sunny S Park, Andrea Conway, Renee N Donahue, David Goldenberg

**Affiliations:** 1 Department of Neural and Behavioral Sciences, The Milton S. Hershey Medical Center, The Pennsylvania State University, Hershey, Pennsylvania, USA; 2 Department of Surgery, Division of Otolaryngology-Head and Neck Surgery, The Milton S. Hershey Medical Center, The Pennsylvania State University, Hershey, Pennsylvania, USA

## Abstract

**Background:**

Carcinoma of the thyroid gland is an uncommon cancer, but the most frequent malignancy of the endocrine system. Most thyroid cancers are derived from the follicular cell. Follicular carcinoma (FTC) is considered more malignant than papillary thyroid carcinoma (PTC), and anaplastic thyroid cancer (ATC) is one of the most lethal human cancers. Opioid Growth Factor (OGF; chemical term - [Met^5^]-enkephalin) and its receptor, OGFr, form an inhibitory axis regulating cell proliferation. Both the peptide and receptor have been detected in a wide variety of cancers, and OGF is currently used clinically as a biotherapy for some non-thyroid neoplasias. This study addressed the question of whether the OGF-OGFr axis is present and functional in human thyroid follicular cell - derived cancer.

**Methods:**

Utilizing human ATC (KAT-18), PTC (KTC-1), and FTC (WRO 82-1) cell lines, immunohistochemistry was employed to ascertain the presence and location of OGF and OGFr. The growth characteristics in the presence of OGF or the opioid antagonist naltrexone (NTX), and the specificity of opioid peptides for proliferation of ATC, were established in KAT-18 cells. Dependence on peptide and receptor were investigated using neutralization studies with antibodies and siRNA experiments, respectively. The mechanism of peptide action on DNA synthesis and cell survival was ascertained. The ubiquity of the OGF-OGFr axis in thyroid follicular cell-derived cancer was assessed in KTC-1 (PTC) and WRO 82-1 (FTC) tumor cells.

**Results:**

OGF and OGFr were present in KAT-18 cells. Concentrations of 10^-6 ^M OGF inhibited cell replication up to 30%, whereas NTX increased cell growth up to 35% relative to cultures treated with sterile water. OGF treatment reduced cell number by as much as 38% in KAT-18 ATC in a dose-dependent and receptor-mediated manner. OGF antibodies neutralized the inhibitory effects of OGF, and siRNA knockdown of OGFr negated growth inhibition by OGF. Cell survival was not altered by OGF, but DNA synthesis as recorded by BrdU incorporation was depressed by 28% in OGF-treated cultures compared to those exposed to sterile water. The OGF-OGFr axis was detected and functional in PTC (KTC-1) and FTC (WRO 82-1) cell lines.

**Conclusion:**

These data suggest that OGF and OGFr are present in follicular-derived thyroid cancers, and that OGF serves in a tonically active inhibitory manner to maintain homeostasis of cell proliferation. These results may provide a biotherapeutic strategy in the treatment of these cancers.

## Background

Thyroid cancer accounts for over 37,000 persons in the U.S. annually, but only 1,600 deaths [1]. Five to 10% of patients with thyroid cancer eventually will die of their disease. It is one of the few malignancies that are more common in females than males (M/F sex ratio, 0.36) [2]. While most thyroid cancers are differentiated and readily treated with ionizing radiation and/or surgery [3,4], cell type is an important determinant of prognosis in thyroid cancer. Thyroid neoplasms arising from follicular cells show a broad range of overlapping clinical and cytologic features [5,6]. Papillary (PTC), follicular (FTC), and anaplastic (ATC) thyroid carcinomas arise from endodermally derived follicular cells present in the thyroid gland. FTC generally comprises about 15% of all diagnosed thyroid cancers and is usually a more aggressive form of cancer than the more common papillary type. Despite its well-differentiated characteristics, FTC may be overtly invasive. In fact, FTC may spread easily to other organs. Life expectancy of affected patients is related to their age. Two to 4% of all thyroid cancers are ATC. ATC generally occurs in a setting of previous thyroid pathology (e.g., preexisting FTC or PTC). The overall 5-year survival rate for ATC is reportedly less than 8%, and most patients do not live longer than a few months after diagnosis. Death usually occurs as a result of local extension and airway compromise or complications from lung metastasis. Treatment is palliative, with a combination of radio- and chemotherapy and tracheostomy tube placement. Surgery is reserved for early tumors without significant extension or local invasion [7,8]. Despite preclinical studies exploring new therapies targeted to molecular pathways [9-13], more effective therapeutic approaches are needed.

The opioid growth factor (OGF), chemically termed [Met^5^]-enkephalin, is an endogenous opioid peptide that is an important regulator in the onset and progression of a variety of human cancers [14-19]. OGF interacts with the OGF receptor (OGFr) to delay the G_1_/S interface of the cell cycle by modulating cyclin-dependent kinase inhibitory (CKI) pathways [20-23]. Attenuation of the OGF-OGFr axis in cancer cells through: i) disruption of OGF-OGFr interfacing by continuous exposure to opioid antagonists (e.g., naltrexone, NTX) [14,17,19], ii) neutralization of OGF by antibodies to the peptide [14,24], or iii) a decrease in OGFr by antisense cDNA or siRNA for OGFr [24,25], stimulates cell proliferation. An increase in OGF-OGFr activity in cancer cells by i) addition of exogenous OGF [14-19], ii) treatment with imidazoquinoline compounds such as imiquimod and resiquimod [24], or iii) transfection of sense cDNA for OGFr [26-29], depresses cell proliferation.

Recently, OGF and OGFr have been detected by immunohistochemistry in surgical samples of non-medullary thyroid cancer including papillary, follicular, and anaplastic, as well as thyroid tissue from patients with non-malignant disease [30]. Moreover, receptor binding data in this report showed that these specimens revealed specific and saturable binding for the nuclear associated OGFr. These results suggest that the OGF-OGFr axis is present in malignant and non-malignant human thyroid tissue.

The present investigation explored the question of whether the OGF-OGFr axis is present and functions in thyroid follicular cell-derived cancer, including the most deadly and untreatable form of thyroid cancer - ATC. Using human ATC, PTC, and FTC cell lines, each previously verified by single nucleotide polymorphism array analysis, the OGF-OGFr axis was identified and characterized functionally. In addition, the specificity of both OGF and OGFr in regulating cell proliferation was determined and mechanisms of peptide action elucidated.

## Methods

### Cell lines and cell proliferation assays

The ATC cell line, KAT-18, was generously provided by Dr. K.B. Ain, The University of Kentucky, Lexington, KY) and the FTC cell line, WRO 82-1, was established by Dr. G. Juillard (University of California at Los Angeles, CA). The poorly differentiated papillary thyroid carcinoma cell line, KTC-1 [31], was a generous gift of Dr. James A. Fagin (Memorial Sloan Kettering Cancer Center, New York, NY). The KAT-18 cells were grown in phenol-red free RPMI 1640 media (GIBCO/Invitrogen, Carlsbad, CA), and the WRO 82-1 and KTC-1 cells were grown in standard RPMI 1640 media supplemented with 1% non-essential amino acids. All media were supplemented with 10% fetal calf serum, 2 mM L-glutamine, 1.2% sodium bicarbonate, and antibiotics (5,000 Units/ml penicillin, 5 mg/ml streptomycin, 10 mg/ml neomycin). The cell cultures were maintained in a humidified atmosphere of 5% CO_2 _at 37°C. All cell lines were free of *Mycoplasma *and confirmed by single nucleotide polymorphism array analysis to be unique follicular cell-derived thyroid cancer cell lines [32].

Cells were seeded at equivalent amounts into 6- or 24-well plates (Falcon) and counted 24 h later (time 0) to determine plating efficiency. OGF or other compounds were added daily; an equivalent volume of sterile water (3 μl) was added to control wells (Co). Media and compounds were replaced daily. Cells were harvested by trypsinization with 0.25% trypsin/0.53 mM EDTA, centrifuged, and counted with a hemacytometer. Cell viability was determined by trypan blue staining. At least two aliquots per well, and 2-4 wells/treatment, were counted for each experiment. Two to five experiments were conducted for each measure performed.

### DNA synthesis, apoptosis and necrosis

The effects of OGF on DNA synthesis (BrdU incorporation), apoptosis (TUNEL), and necrosis (trypan blue) were evaluated. To examine DNA synthesis, KAT-18 cells were seeded onto 22 mm diameter coverglasses placed in 6-well plates (3 × 10^3 ^cells/coverglass), and treated with compounds for 72 h; media and drugs were replaced daily. Three h prior to fixing cells, 30 μM BrdU (Sigma Chemicals, Indianapolis, IN) was added to cultures. Cells were rinsed, fixed in 10% neutral buffered formalin for 10 min, and stained with antibodies to BrdU (anti-BrdU-BOD, Roche/Invitrogen). At least 1000 cells/treatment using at least 2 coverglasses/treatment were counted, and the number of positive cells recorded. Labeling indexes were calculated as the number of positive stained cells divided by total cells.

To detect late-stage apoptosis, TUNEL assays (Trevigen, Gaithersburg, MD) were performed on KAT-18 cells seeded on coverglasses in 6-well plates and treated with OGF or sterile water beginning 24 h later; drugs and media were replaced daily. Cells were harvested after 72 h of drug treatment, and TUNEL assays conducted according to manufacturer's recommendations.

Necrosis was recorded as the number of positive trypan blue cells in any cell growth assay.

### Immunohistochemistry

To examine for the presence of OGF and OGFr, log-phase KAT-18, KTC-1, and WRO 82-1 cells were plated onto 22 mm round coverglasses. After 72 h in culture, cells were fixed and permeabilized in 95% ethanol and acetone at -20°C, and processed according to previously published procedures [14]. Cells were incubated with ammonium sulfate purified anti- [Met^5^]-enkephalin IgG (CO172) [33] or anti-OGFr-IgG (BO344) [33,34] antibodies diluted 1:250 in Sorenson's phosphate buffer containing 1% normal goat serum and 0.1% Triton X-100 for 18 h at 4°C, washed and incubated with goat anti-rabbit IgG (1:1000) conjugated to rhodamine prior to viewing with fluorescence microscopy. Coverglasses containing cells that were incubated with secondary antibody only served as controls.

### OGFr binding assays

Binding assays for OGFr were performed with procedures described earlier [26,27]. In brief, log phase KAT-18 cells were harvested, and the nuclear fraction isolated through sucrose gradient centrifugation. Binding saturation isotherms were determined by specific binding of custom-synthesized [^3^H]- [Met^5^]-enkephalin (Perkin Elmer-New England Nuclear; 52.7 Ci/mmol). Independent assays were performed 2 to 4 times in duplicate.

### Specificity of endogenous OGF

To determine the specificity of endogenous OGF, log-phase KAT-18 cells were exposed to a polyclonal antibody to OGF (1:200; CO172) [33] or to pre-immune rabbit serum (1:200) as a control. Serum and media were changed daily, and cells counted after 72 h. Cell viability was determined by trypan blue staining. At least two aliquots per well, and 2-6 wells/treatment, were counted.

### siRNA knockdown of OGFr: Northern analyses and semi-quantitative immunohistochemistry

The OGFr-targeted siRNAs (antisense:5'-uagaaacucagguuuggcg-3'; sense: 5'-cgccaaaccugaguuucua-3') were designed and obtained as ready-annealed, purified duplex probes from Ambion (Austin, TX) [25]. For transfection, 5 × 10^4 ^KAT-18 cells per well were seeded in 6-well plates containing 1 ml of serum-containing media without antibiotics. In each well, 20 nM OGFr-siRNA or control siRNA solutions in serum-free media were added. Cells were incubated for 4 h at 37°C prior to the addition of OGF or NTX (10^-6 ^M). Cultures were incubated an additional 20 h, and then 1 ml fresh complete media either lacking or containing OGF or NTX was added. At 72 h cells were collected for computing growth. Two independent experiments were conducted. The scrambled siRNAs were purchased from Ambion.

Some cultures were transfected with siRNAs and then harvested 72 h later for Northern analysis. Total RNA was extracted using the Paris Kit (Ambion), separated on an agarose gel, and mRNA transferred to nylon membrane (Immobilon, Bio-Rad Laboratories, Hercules, CA). Membranes were probed with ^32^P-dCTP- OGFr cDNA. To determine equal loading, blots were stripped and reprobed with radiolabeled GAPDH and optical density of each band was determined by densitometry and analyzed by QuickOne (Bio-Rad Laboratories). Each value was normalized to GAPDH from the same blot.

KAT-18 cells were seeded onto coverglasses, transiently transfected with OGFr-siRNA, and 72 h later stained with polyclonal antibodies to OGFr in order to detect the level of protein knockdown. OGFr protein levels were assessed by semi-quantitative immunocytochemistry whereby images were taken at the same exposure time with special care not to photobleach samples. The mean intensity of staining was determined for approximately 50 cells/group.

### Chemicals

The following compounds were obtained from the indicated sources: [Met^5^]-enkephalin, [Leu^5^]-enkephalin, [D-Pen^2,5^]-enkephalin (DPDPE), [D-Ala^2^, MePhe^4^, Glycol^5^]-enkephalin (DAMGO), -endorphin, naltrexone (NTX), naloxone, dynorphin A1-8 (Dynorphin), morphine sulfate, endomorphin 1 (Endo 1), endomorphin 2 (Endo 2), Sigma (St. Louis, MO); U69,583, Upjohn Diagnostics (Kalamazoo, MI).

### Statistical analyses

All calculations were performed with GraphPad Prism software. Cell numbers and intensity measurements were analyzed using two-tailed t-tests or analysis of variance with subsequent comparisons made using Newman-Keuls tests.

## Results

### OGF and OGFr are present in anaplastic thyroid cells

**Figure 1:**
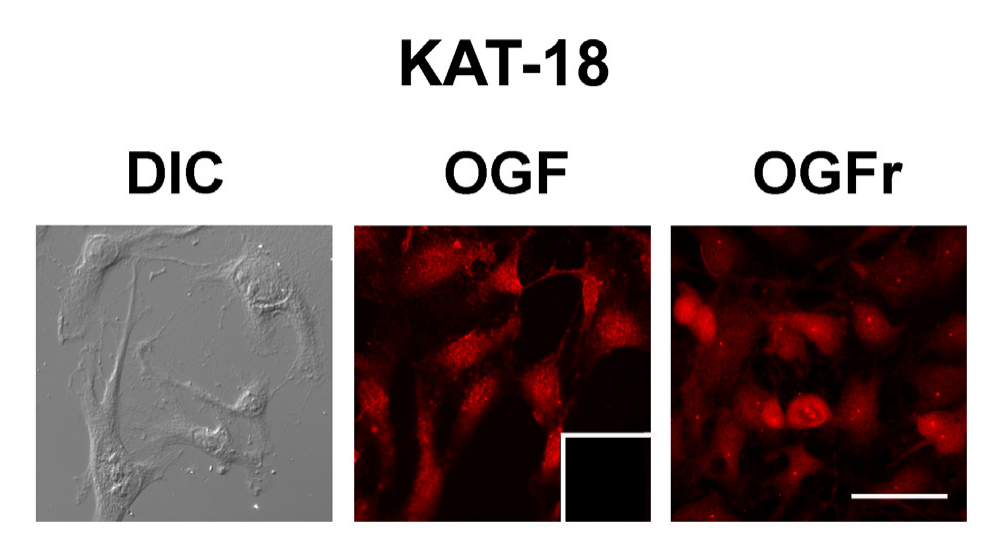
**The presence and distribution of OGF and OGFr in human anaplastic thyroid cancer cells**. Photomicrographs of log-phase KAT-18 cells visualized with differential interference or stained with antibodies to [Met^5^]-enkephalin (OGF) or OGFr. Immunoreactivity was associated with the cytoplasm and a speckling of stain was noted in cell nuclei; no staining was observed with secondary antibodies only (inset). Scale bar = 10 μm.

Antibodies to OGF and to OGFr were used with immunohistochemistry to determine the presence and location of this growth-related peptide and its receptor in cultures of KAT-18 (Fig. 1). Immunoreactive OGF and OGFr were localized to the cytoplasm with speckling noted in the nucleus. No staining was recorded in control specimens processed with secondary antibody only.

Receptor binding analyses of KAT-18 revealed specific and saturable binding, with a one-site model of binding. The binding capacity (B_max_) was 9.3 ± 0.6 fmol/mg protein and binding affinity (K_d_) was 3.5 ± 0.6 nM.

### OGF depresses growth of anaplastic thyroid cells in a dose-dependent, receptor mediated, and reversible manner

**Figure 2:**
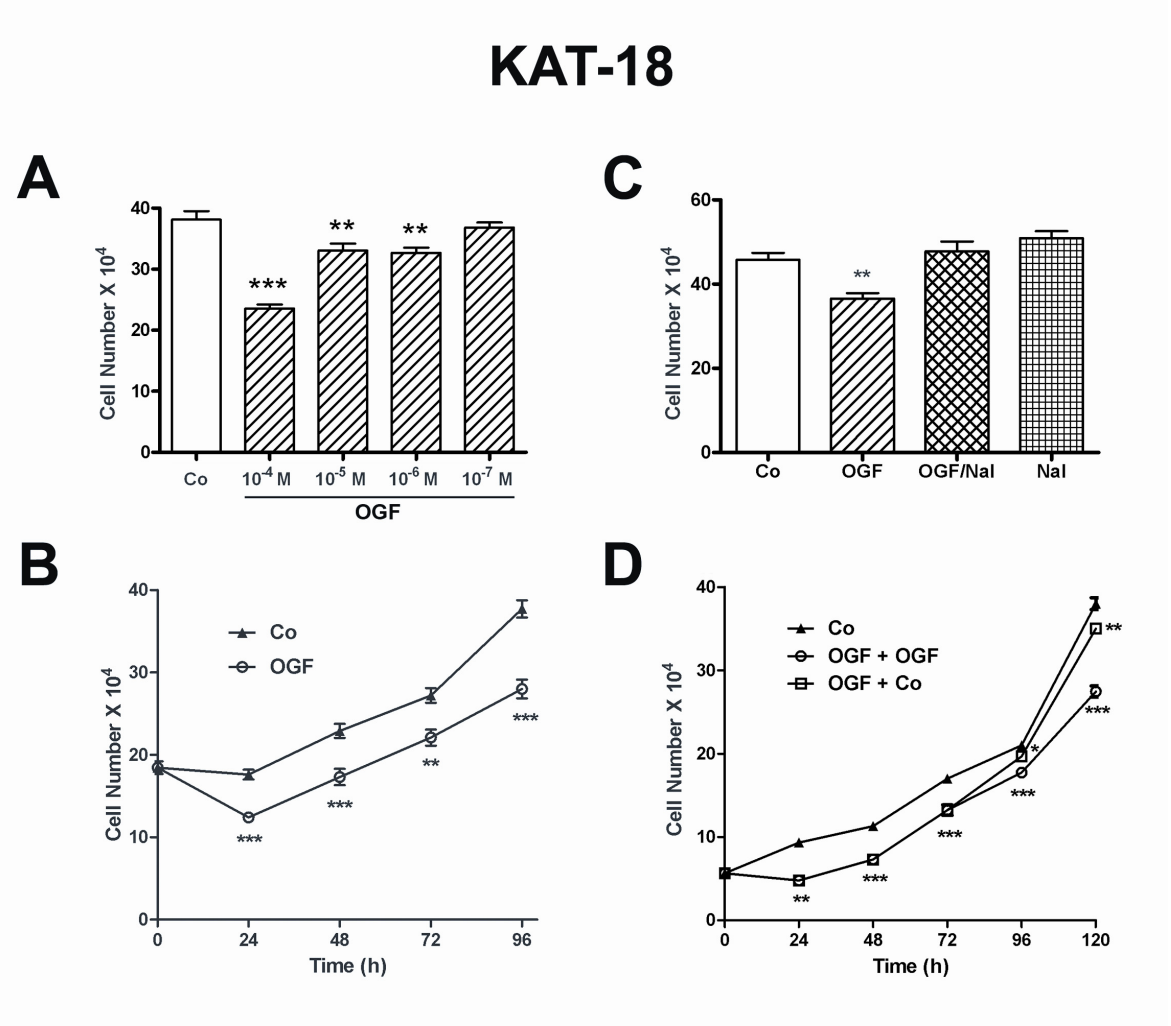
**OGF inhibits growth of human anaplastic thyroid cancer cells in a dose-dependent, receptor-mediated, and reversible manner**. (A) Growth of KAT-18 cells subjected to various concentrations of opioid growth factor (OGF) of sterile water (Co) for 72 h. (B) Growth of KAT-18 cells treated with OGF (10^-6 ^M) or an equivalent volume of sterile water (Co) over a 96-h period of time. (C) Opioid receptor mediation of the growth inhibitory effects of OGF in KAT-18 cells. Cell cultures were subjected to OGF (10^-6 ^M), the opioid antagonist naloxone (Nal; 10^-6 ^M), OGF and Nal, or sterile water (Co) for 72 h. (D) Reversibility of the growth inhibitory effects on KAT-18 cells treated with 10^-6 ^M OGF or sterile water (Co). At 72 h, one-half of the culture plates continued to receive OGF for an additional 48 h, and one-half of the plates were treated with sterile water for 48 h. Control cultures received sterile water throughout the 120 h. For all experiments, data represent mean SE for at least 2 aliquots/well from 2 wells/group. Two to five experiments were conducted for each measure. Significantly different from respective controls at p < 0.05 (*), p < 0.01 (**), p < 0.001 (***).

The effects of OGF on KAT-18 cells were evaluated in dose-response studies (Fig. 2A). At 72 h, dosages of 10^-4^, 10^-5^, and 10^-6 ^M OGF reduced cell number by 38%, 13%, and 14%, respectively, from control levels. Exposure to 10^-7 ^M OGF had no significant effect on cell number in comparison to control values. Using a dose of 10^-6 ^M OGF, cultures were reduced in cell number by 13% to 30% from control cultures (Fig. 2B) over a 96 h period of time.

In order to determine whether the inhibitory effect of OGF was mediated by an opioid receptor, KAT-18 cells were grown in the presence of OGF and the short-acting opioid receptor antagonist naloxone (10^-6 ^M; Nal) (Fig. 2C). Cell number was reduced by 20% in cultures treated with 10^-6 ^M OGF compared to control levels, whereas cultures receiving both OGF and naloxone or naloxone alone had no change in cell number.

To address the question of whether the effects of OGF on cell number persisted after removal of the drug, a study was performed to determine if the inhibitory effects of OGF could be reversed (Fig. 2D). At 72 h after exposure to 10^-6 ^M OGF, wherein a reduction of 22% in cell number was recorded compared to control levels, cell cultures grown in media replenished without OGF were reduced by 6% and 8%, respectively, from control cultures at 96 and 120 h. In contrast, cultures maintained on OGF were depressed 16% and 28% at 96 and 120 h, respectively, from cells subjected to sterile water. At 120 h, cells released from OGF exposure were 21% greater in number than those continuing to receive OGF; this difference was statistically significant at p < 0.01. Calculation of the increase in number of cells/h for log-phase growth (between 96 h and 120 h after seeding) indicated that the controls grew at a rate of 7,083 cells/h in contrast to a rate of 6,375 cells/h for the group exposed to OGF and subsequently incubated in compound-free media. Cultures continuing to be maintained on OGF (i.e., exposure to OGF for 120 h), increased at a rate of 4,083 cells/h during this period.

### The endogenous opioid specific for growth inhibition of anaplastic thyroid cells is OGF

**Figure 3:**
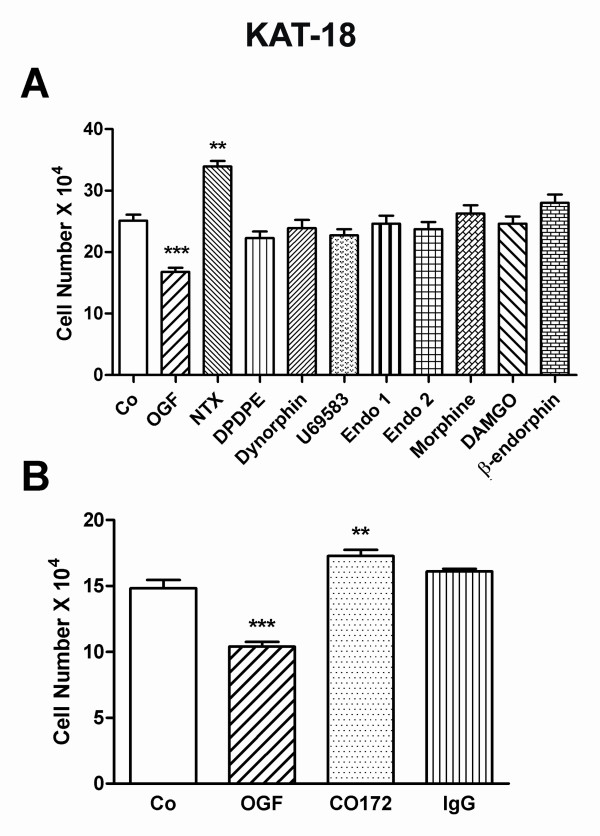
**OGF is the specific opioid peptide involved in the growth inhibition of human anaplastic thyroid cancer cells**. (A) The effects of various endogenous and exogenous opioids (10^-6 ^M) or sterile water (Co) on KAT-18 cell number. (B) Cells were treated with an antibody specific for OGF (CO172), pre-immune serum (IgG), exogenous OGF (10^-6 ^M), or sterile water (Co). Cell number was measured at 72 h. Data for both experiments represent mean SE for at least 2 aliquots/well from 2 wells/group. Three experiments were conducted for each measure. Significantly different from respective controls at p < 0.01 (**) or p < 0.001 (***).

Although OGF inhibits the growth of ATC cells, a variety of other endogenous and exogenous opioids and opioid antagonists were tested for their ability to alter cell number (Fig. 3A). Log phase cultures of KAT-18 cells were exposed to 10^-6 ^M concentrations, a dosage of OGF shown to significantly reduce growth from controls at 72 h of exposure. Compounds were chosen as those that were specific agonists to classical opioid receptors (μ, δ, κ): DAMGO, morphine, endomorphin 1, endomorphin 2, DPDPE, dynorphin A1-8, U69683, and β-endorphin. Only OGF altered cell proliferation significantly; OGF decreased cell number by 33% from controls (Fig. 3A).

The effect of the general opioid antagonist NTX on the growth of human ATC cells showed that this drug increased cell number by 35% from control values after 72 h in culture (Fig. 3A).

The specificity of OGF action was further investigated by adding polyclonal antibodies to OGF to determine the effects of neutralization of endogenous OGF on cell number (Fig. 3B). Cultures were treated with 10^-6 ^M OGF, OGF antibody CO172, or IgG. Cultures exposed to the OGF antibody had 16% more cells than control cultures; cultures treated with sterile water and those receiving IgG had a similar number of cells.

### Silencing of OGFr in human anaplastic thyroid cells attenuates the inhibitory action of endogenous and exogenous OGF

**Figure 4:**
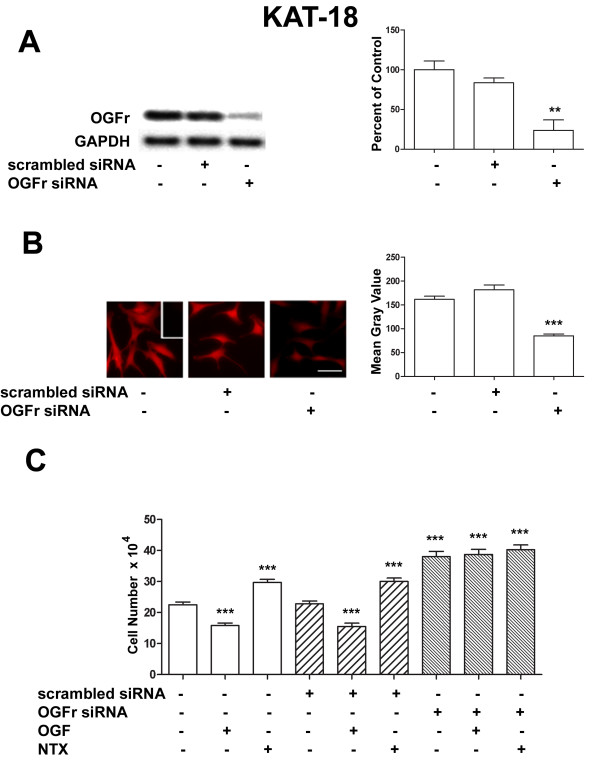
**OGFr is required for OGF's inhibitory action on growth**. (A) Northern blot analysis demonstrating the specificity of the OGFr siRNA in KAT-18 cells. Log phase cells were transfected for 24 h with either scrambled siRNA or OGFr siRNA. Quantitative densitometry (percent of OGFr/GAPDH ratio) represent mean ± SEM for 2 blots from independent experiments. Significantly different from non-transfected cultures at p < 0.01 (**). (B) Immunocytochemical detection of OGFr demonstrating the knockdown of receptor protein. Photomicrographs of cultures transfected with OGFr siRNA, scrambled siRNA, or untransfected and stained with OGFr antibodies. Data (mean gray value) represent mean ± SEM for fluorescence measurements of at least 50 cells. Significantly different from non-transfected cells and those transfected with scrambled siRNA at p < 0.001 (***). (C) Growth of KAT-18 cultures transfected with OGFr siRNA or scrambled siRNA for 24 h and treated with either OGF (10^-6 ^M), NTX (10^-6 ^M), or an equivalent volume of sterile water for an additional 48 h; compounds and media were changed daily. Values represent mean ± SE cell counts for 2 aliquots/well and 2 wells/treatment from three experiments. Significantly different at p < 0.001 (***) from cultures that were not transfected as well as cells that were transfected with scrambled siRNA.

The requirement of the OGF receptor for OGF action on cell proliferation was examined at the molecular level using siRNA technology. Transfection of KAT-18 cells with OGFr siRNA effectively knocked down OGFr activity (Fig. 4). Northern blot analyses revealed that transfected KAT-18 cells had less than 25% OGFr relative to cells treated with vehicle only (Fig. 4A); cells exposed to scrambled siRNA were comparable to those receiving vehicle only. Immunocytochemical staining of cells transfected with siRNA-OGFr was assessed semi-quantitatively and revealed a 47% decrease in OGFr protein expression relative to both untransfected cells and those transfected with scrambled siRNA (Fig. 4B). Silencing of OGFr activity using siRNA technology resulted in functional changes as indicated by cell number. KAT-18 cells transfected with OGFr siRNA had approximately 70% more cells that cultures exposed to vehicle only, as well as those transfected with scrambled siRNA (Fig. 4C). Addition of exogenous OGF had no effect on cells transfected with OGFr siRNA, but cell number was reduced by 30% in both untransfected cultures and those transfected with scrambled siRNAs. KAT-18 cells transfected with OGFr siRNA and treated with OGF or sterile water did not differ in cell number from each other, and all differed significantly from wildtype cultures and those transfected with scrambled siRNAs.

NTX is a general opioid receptor antagonist that upregulates cell number. To inquire as to whether NTX is dependent on OGFr, cells treated with OGFr siRNA were exposed to 10^-6 ^M NTX. Untreated and scrambled siRNA cultures exposed to NTX increased in cell number by 32% in comparison to wildtype cells receiving vehicle. NTX added to cultures receiving OGFr siRNA increased by 70% in contrast to wildtype cells, and these cultures treated with OGFr siRNA and exposed to NTX did not differ from cells receiving OGFr siRNA plus vehicle.

### OGF alters DNA synthesis but not apoptosis or necrosis

**Figure 5:**
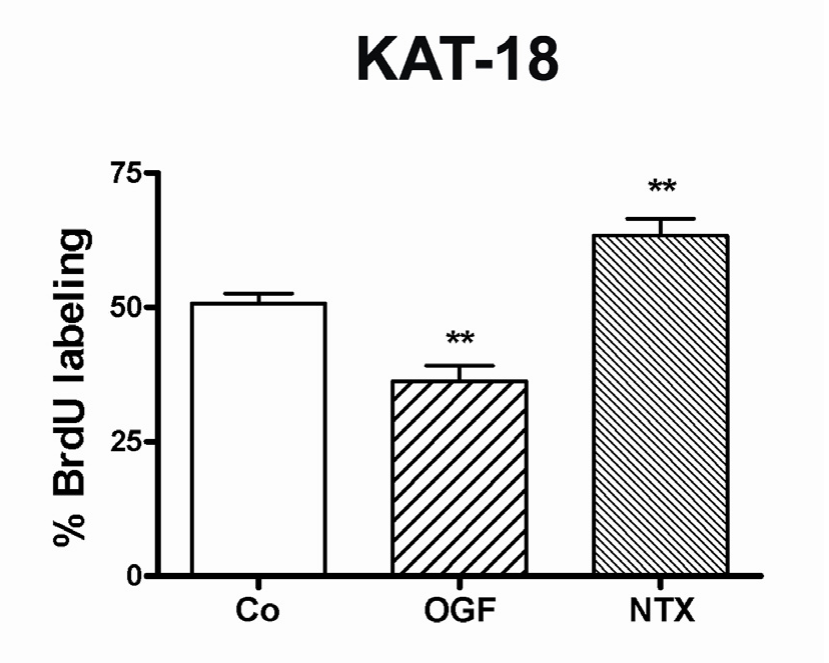
**DNA synthesis is a mechanism of OGF inhibition**. Human KAT-18 anaplastic thyroid cancer cells were treated with 10^-6 ^M OGF or NTX, or an equivalent volume of sterile water (Co). Data represent the percent BrdU positive cells (mean ± SE). Significantly different from controls at p < 0.01 (**).

To examine the mechanism by which OGF reduces human thyroid cancer cells *in vitro*, DNA synthesis of KAT-18 cultures exposed to OGF, NTX, or sterile water was measured (Fig. 5). The proportion of BrdU labeled cells in cultures exposed to OGF was 28% less than that in cultures receiving sterile water, while NTX increased DNA synthesis by 25% relative to controls after 72 h of drug exposure.

No differences in the number of apoptotic or necrotic cells were noted between groups receiving sterile water, OGF, or NTX (data not shown). In fact, relative to positive control samples for apoptosis, apoptosis (and necrosis) in cultures treated with OGF, NTX, or saline was negligible.

### The OGF-OGFr axis is present and functions in both papillary and follicular thyroid cancer cell lines

**Figure 6:**
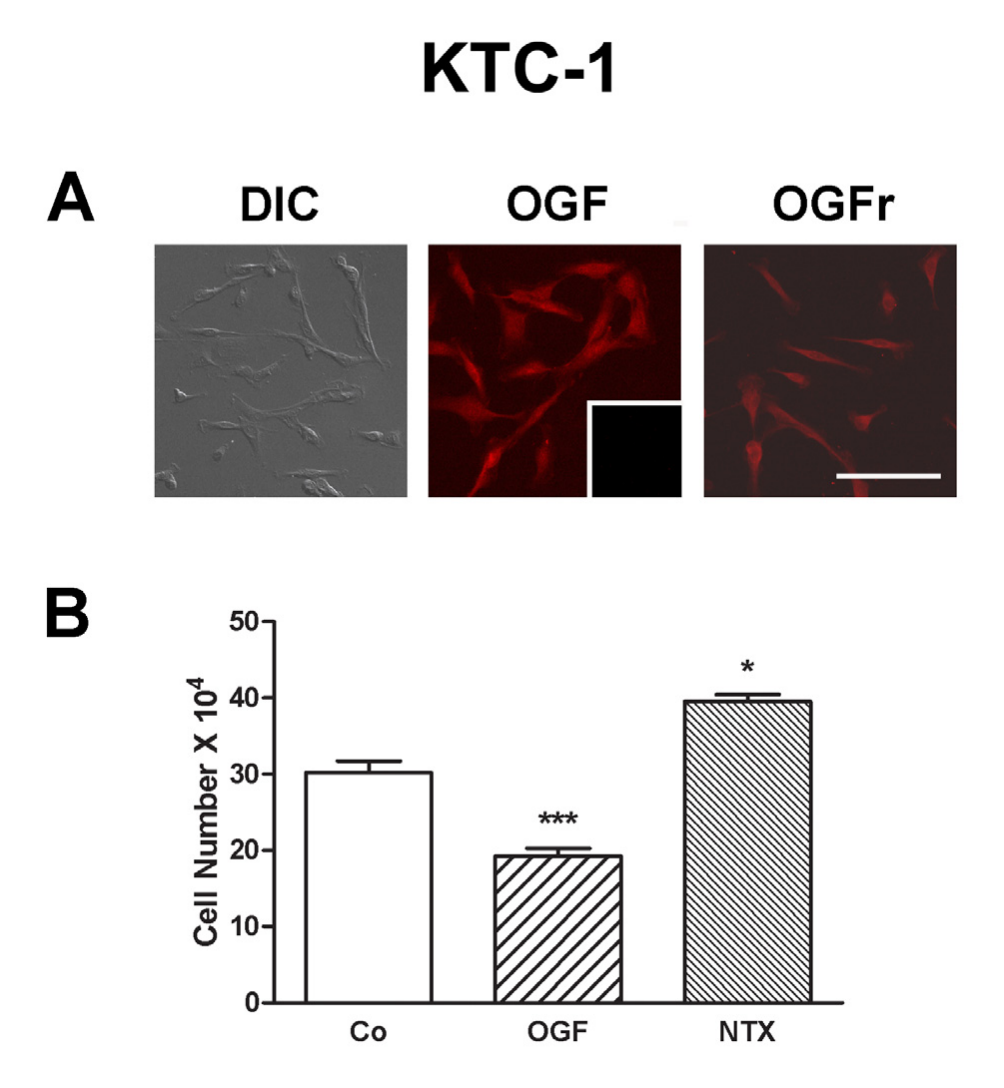
**The presence and distribution of OGF and OGFr in human papillary thyroid cancer cells**. (A) Photomicrographs of log-phase KTC-1 cells visualized with differential interference (DIC) or stained with antibodies to [Met^5^]-enkephalin (OGF) or OGFr. Immunoreactivity was associated with the cytoplasm and a speckling of stain was noted in cell nuclei; no staining was detected with secondary antibodies only (inset). Scale bar = 10 μm. (B) KTC-1 cells were treated for 72 h with 10^-6 ^M OGF or NTX, or sterile water (Co). Values represent mean ± SE for cells counted from 2 wells/treatment group from three experiments. Significantly different from controls at p < 0.05 (*) or p < 0.001 (***).

**Figure 7:**
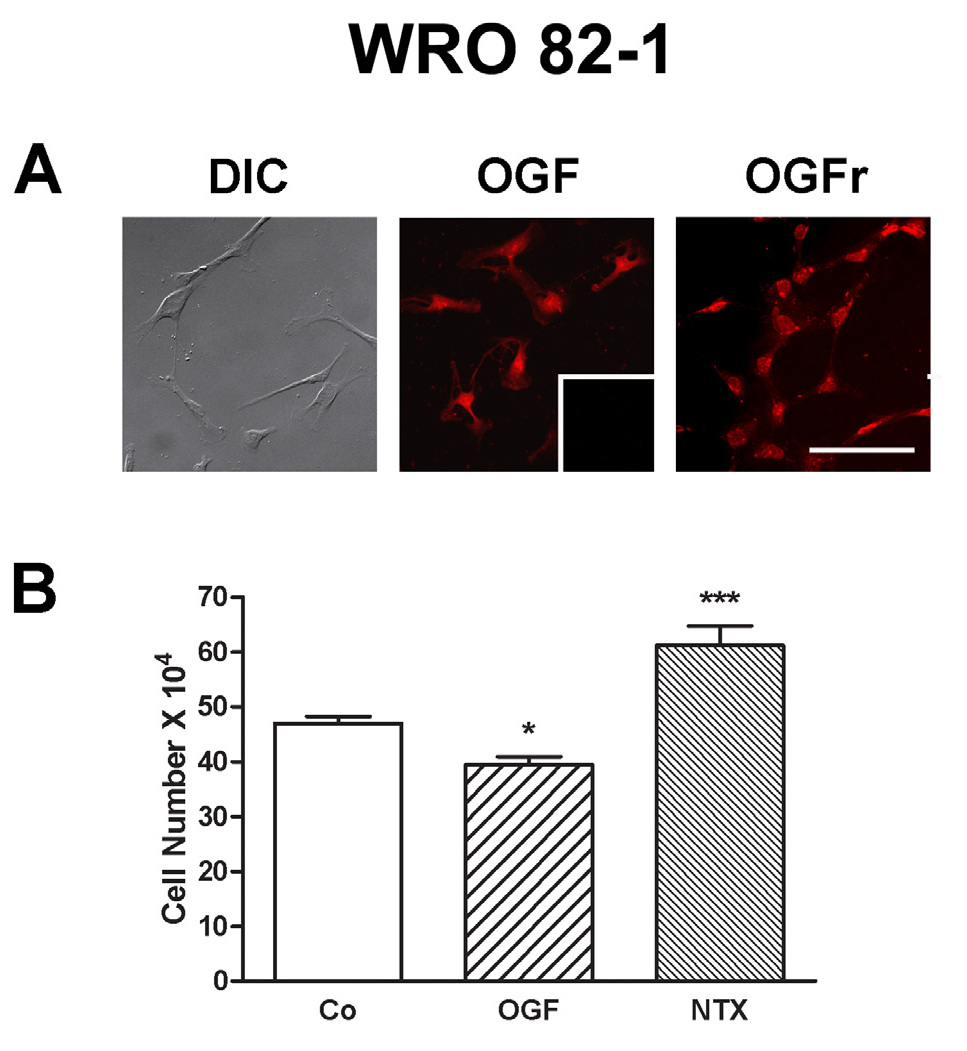
**The presence and distribution of OGF and OGFr in human follicular thyroid cancer cells**. (A) Photomicrographs of log-phase WRO 82-1 cells visualized with differential interference (DIC) or stained with antibodies to [Met^5^]-enkephalin (OGF) or OGFr. Immunoreactivity was associated with the cytoplasm and a speckling of stain was noted in cell nuclei; no staining was recorded with secondary antibodies only (inset). Scale bar = 10 μm. (B) WRO 82-1 cells were treated for 48 h with 10^-6 ^M OGF or NTX, or sterile water (Co). Values represent means ± SE for cells counted from 2 wells/treatment group from three experiments. Significantly different from controls at p < 0.05 (*) or p < 0.001 (***).

OGF and OGFr were detected in both KTC-1 (PTC) (Fig. 6A) and WRO 82-1 (FTC) (Fig. 7A) cell lines. After 72 h of treatment with OGF (10^-6 ^M) KTC-1 and WRO 82-1 cells were reduced by 31% and 17% from respective control levels (Figs. 6B, 7B). Exposure of KTC-1 and WRO 82-1 cells for 72 h to 10^-6 ^M concentrations of NTX increased cell number in both cell lines by 30% (Figs. 6B, 7B).

## Discussion

A previous study in our laboratory has demonstrated the presence of OGF and OGFr in human PTC, FTC, and ATC surgical specimens [30]. A critical question arising from this earlier investigation is whether opioids are present and function in thyroid follicular cell - derived cancers. Using a human ATC cell line, KAT-18, in a tissue culture model, and confirming the presence of OGF and OGFr in these human cells under *in vitro *conditions, we report for the first time that opioids do indeed function in these deadly neoplasias, subserving as modulators of cell proliferation. The data reveal that at least one opioid, OGF, exerted a dose-dependent inhibitory action on these ATC cells as measured by changes in cell number. The growth repression recorded was rapid, being observed within 24 h after initiating drug exposure, and continuing in the presence of drug for up to 120 h. The effect of OGF was mediated by opioid receptors, because an opioid antagonist of short duration and low potency, naloxone, blocked the depression in cell acquisition but by itself did not have any influence on cell replication. Moreover, the consequences of OGF on repressing cell proliferation were reversible when OGF-exposed cells were transferred from OGF-containing media to fresh media without compound, suggesting that this peptide acted in a cytostatic rather than toxic manner. Examination of the ubiquity of the OGF-OGFr axis in other types of follicular cell-derived thyroid cancers revealed that both the peptide and receptor were present, and that this biological system functioned as a growth-inhibitory pathway. These data suggest that the OGF-OGFr axis is present and a determinant of thyroid follicular cell-derived cancers in tissue culture, extending similar observations using surgical preparations [30]. Further research is needed to characterize this system on other human PTC, FTC, and ATC cancer cell lines, and to determine if the OGF-OGFr axis can modulate the growth of xenografts with these neoplasias.

Given that OGF is an endogenous opioid peptide, and that this peptide was provided to the cells exogenously in these initial experiments, we raised the question of whether endogenous OGF itself is involved with regulating cell proliferation of thyroid follicular cell-derived cancers. In the first set of studies, we treated ATC cells with the long acting and extremely potent general opioid antagonist, NTX. We reasoned that if endogenous opioids such as OGF repressed cell proliferation, then blockade of opioid peptides from opioid receptors should increase the number of cells above control levels, especially if OGF was continuously secreted and active in its regulatory role. Indeed, administration of NTX increased cell number in these cultures compared to cultures treated with either vehicle or OGF. These data indicate that OGF is tonically active in maintaining a homeostatic equilibrium of proliferation of thyroid follicular cell-derived cancers. In a second series of experiments, the assessment of a variety of synthetic and natural neuropeptides related to opioid receptors (particularly μ, δ, and κ) were tested for their ability to influence cell proliferation. Not one of these ligands that recognized classical opioid receptors had an effect on the proliferative properties of human ATC cells. In a third series of experiments related to the question of opioid specificity, cultures were exposed to an antibody to OGF in order to examine whether this antibody could neutralize OGF action. In fact, treatment with this antibody to OGF resulted in cell proliferation that was elevated from control levels, thereby speaking to the specificity of the endogenous opioid involved with repression of cell replication, as well as affirming and extending the results of experiments with NTX showing that OGF activity in regulating cell number is tonically active. In addition, because OGF is known to be produced and secreted by a variety of cancer and non-cancer cells [17], our findings suggest that this peptide acts in an autocrine and presumably paracrine manner in thyroid follicular cell-derived cancer cells.

An extensive literature shows that OGF interacts with OGFr to regulate cell the proliferation of human and animal cells, normal and cancer [14,15,17-29], implying that the effect on control of cell number by endogenous OGF, as well as exogenously administered peptide, is mediated by this receptor. OGFr has been detected in ATC, FTC, and PTC by immunohistochemistry, and specific and saturable binding of OGF to OGFr was quantitated [30]. However, OGF is [Met^5^]-enkephalin and this opioid peptide is known to bind to classical opioid receptors such as μ, δ, and κ as well [23]. In order to examine the specificity of OGF for OGFr with respect to regulation of cell proliferation, the effect of silencing OGFr using siRNA technology was undertaken. KAT-18 (ATC) cells treated with OGFr siRNA were observed to have an increase in cell number, suggesting that attenuating OGFr compromised endogenously produced OGF. Moreover, exogenously administered OGF which is known to depress cell number in log phase cultures did not have any effect when the cells were transfected with OGFr siRNA. In fact, there were a greater number of cells in cultures treated with OGFr siRNA and exposed to OGF than in cultures given vehicle or scrambled siRNA. An interesting observation in these studies is that although the use of NTX, a general opioid receptor blocking agent, increased cell number, knockdown of OGFr by OGFr siRNA was over twice as effective. These results are consistent with earlier findings using immunoelectron microscopy showing that there is still some OGF-OGFr activity even with treatment of NTX [35], indicating "leakiness" with NTX blockade. That NTX is not the most specific blocker of OGF-OGFr interaction is understandable because the OGF receptor has nucleotide and protein sequences that are not in keeping with classical opioid receptors [23]. Thus, NTX appears to have recognition of OGFr, but does not provide a full blockade of OGF-OGFr interfacing. All of these data support OGFr as the receptor mediating OGF action. Taken together with knowledge that OGF is the peptide involved with modulating cell number of human thyroid follicular cell-derived cancer cells, it is clear that proliferation of these carcinoma cells is dependent on the OGF-OGFr axis.

A decrease in cell number as seen by treatment with OGF could be due to a decrease in cell survival because of programmed cell death, necrosis, and/or a reduction in DNA synthesis and subsequent cell replication. Our data show that neither apoptosis nor necrosis are involved with OGF activity in ATC cells, a result consistent with a previous publication documenting a similar finding in a variety of cancer cells growing in tissue culture [36]. However, DNA synthesis measured over a 3-h period in ATC cells treated with OGF was diminished from control levels by 28, whereas blockade of OGF-OGFr interaction by NTX elevated DNA synthesis by 25%. Consistent with previous studies on head and neck cancers [14,20], these data indicate that the mechanism of OGF involves regulation of cell proliferation. Cheng et al. [21,22] have found that OGF regulates the p16 pathway in human squamous cell carcinoma and the p21 pathway in human pancreatic neoplasia, suggesting that cyclin-dependent inhibitory kinase (CKI) pathways are a target of OGF. Further studies with human thyroid follicular cell-derived cancers are warranted to see if one or both of these CKIs play a role in OGF's regulatory activity of cell proliferation.

A critical question that needs to be addressed in future studies is whether OGF has efficacy in modulating the incidence and progression of human ATC, PTC, and FTC *in vivo*. Preclinical experiments are needed to establish whether this agent can alter the course of these lethal neoplasias. If successful, then the role of OGF in the etiology and pathogenesis of these cancers will be an exciting avenue to pursue and studies using OGF as an antitumor agent in the clinic are warranted.

## Conclusion

These data demonstrate that the OGF-OGFr axis is present in follicular-derived thyroid cancers, and that OGF is a tonically active inhibitory peptide that serves in a receptor-mediated manner to repress proliferation of these thyroid cancer cells. These results support further consideration on the use of OGF as a biotherapeutic strategy in the treatment of follicular-derived thyroid cancers.

## Abbreviations

OGF: opioid growth factor; OGFr: opioid growth factor receptor; ATC: anaplastic thyroid cancer; PTC: papillary thyroid cancer; FTC: follicular thyroid cancer

## Competing interests

PJM and ISZ hold patents (individually and with Penn State University) on both OGFr and use of OGF to regulate growth of pancreatic and colorectal cancer.

DG, SSP, RND, and ABC have no competing interests.

## Authors' contributions

PJM, DG, and ISZ participated in the conception of the project, and overall analysis and interpretation of data. In addition, PJM and ISZ were involved in the design and drafting of the manuscript. SSP, ABC, RND, and PJM carried out acquisition of the data and statistical analyses. All authors have approved the final manuscript.

## Pre-publication history

The pre-publication history for this paper can be accessed here:

http://www.biomedcentral.com/1471-2407/9/369/prepub
